# Competition between bound and free peptides in an ELISA-based procedure that assays peptides derived from protein digests

**DOI:** 10.1186/1477-5956-4-12

**Published:** 2006-05-31

**Authors:** Ori Braitbard, Hava Glickstein, Janette Bishara-Shieban, Umberto Pace, Wilfred D Stein

**Affiliations:** 1 Biological Chemistry, Silberman Institute of Life Sciences, Hebrew University, Jerusalem, Israel; 2 MDR Tests Ltd, 28 Pierre Koenig St, Jerusalem, Israel

## Abstract

**Background:**

We describe an ELISA-based method that can be used to identify and quantitate proteins in biological samples. In this method, peptides in solution, derived from proteolytic digests of the sample, compete with substrate-attached synthetic peptides for antibodies, also in solution, generated against the chosen peptides. The peptides used for the ELISA are chosen on the basis of their being (i) products of the proteolytic (e.g. tryptic) digestion of the protein to be identified and (ii) unique to the target protein, as far as one can know from the published sequences.

**Results:**

In this paper we describe the competition assay and we define the optimal conditions for the most effective assay. We have performed an analysis of the kinetics of interaction between the four components of the assay: the plastic substratum to which the peptide is bound, the bound peptide itself, the competing added peptide, and the antibody that is specific for the peptide and we compare the results of theoretical simulations to the actual data in some model systems.

**Conclusion:**

The data suggest that the peptides bind to the plastic substratum in more than one conformation and that, once bound, the peptide displays different affinities for the antibody, depending on how it has bound to the plate

## Background

We have developed the Peptidomatrix, a method for the quantitative analysis of proteins in biological samples. The method's main thrust is that peptides, derived from proteolytic digestion of all the proteins in the sample, rather than the proteins themselves, are assayed. The assay is a competition ELISA and in order to obtain quantitative data a calibration curve with synthetic peptides is run in every experiment. In a related paper (Braitbard et al., submitted) we present the application of Peptidomatrix to the detection of four membrane proteins in cell culture and human lymphocytes.

Here we present a study of the kinetics of interaction between the four components of the Peptidomatrix: (1) The plastic substratum to which the peptide is bound; (2) the bound peptide itself; (3) the competing added peptide; and (4) the antibody that is specific for the peptide [1,2].

There have been very many studies, both experimental and theoretical, of ELISA assays using antibody-peptide interactions. In the majority of these studies the antibodies themselves are bound to the plastic substratum [1-13]. These papers had aims that were somewhat different from those with which we are concerned. Some studied the influence of the length of the epitope on the affinity [5], others the ability to design vaccine according to the nature of the peptide [6], the identification of antibodies in sera [7] or understanding the structure of the subunit of the protein [8]. We have been able to find very few examples in which a peptide antigen is bound to the solid support. [2,9-11]. Again, these papers concern themselves with issues such as the chemical nature of the binding reaction, [11], which are not coincident with ours, and in none of these is a full theoretical treatment presented. Other studies involve the use of the BIAcore technology [2,10] and, although very interesting in themselves, do not help us directly in our studies that use a plastic culture dish substratum. We have attempted to fill this gap as far as we are able.

To understand the interaction between the various components of our competitive ELISA, one should note that what we are concerned with is not the intrinsic affinity between ligand and antibody but, instead, the avidity. Affinity is the measure of the intrinsic strength of the binding of an epitope to an antibody. Avidity is the operational measure of the overall stability of the complex between antibody and antigen and this is governed by three major factors: (1) The intrinsic affinity of the antibody for the epitope; (2) the valency of the combination between antibody and antigen and (3) the geometric arrangement of the interacting components [12].

To calculate the avidities in our competitive ELISA system we have to consider the kinetics of interaction between the four components of the assay: the plastic substratum to which the peptide is bound, the bound peptide itself, the competing added peptide, and the antibody that is specific for the peptide.

The special structures of the peptides and the different possibilities by which they become bound to the plastic substratum influence the avidity, and distinguish it from other antigens such as whole proteins or peptides that are bound to a carrier.

Binding between antibodies and their specific polypeptide ligands is conventionally analyzed in terms of operationally measured, hyperbolic binding isotherms. Binding between molecules and the plastic substratum of ELISA plates is likewise analyzed in general in terms of adsorption isotherms. We combined these two approaches and set up models in which the adsorption to the plate is a simple isotherm or a more complex one. Comparison with the experimental data suggests that the peptides bind to the plastic substratum with more than one modality and that, once bound, the peptide displays different affinities for the antibody depending on how it is bound to the plate.

## Results

The Peptidomatrix assay uses peptides that are chosen as being (i) specific for a target protein and (ii) amongst the products of tryptic digestion of that protein. We subjected the membrane protein transporters MXR (or BCRP i.e. ABCG2) and MRP1 (ABCC1), and the alpha chain of Na,K_ATPase (ATP1A1) to a virtual tryptic digestion and selected all the peptides of length 7 to 15 amino-acids. Each one of these peptides was analyzed using the BLAST program (see methods). The desirable peptide contains only matches that are 5 amino acids or shorter, and a minimal number of them. The peptides chosen are listed in Table 1.

**Table 1:** Peptides and antibodies used throughout this study. Note that all these peptides have a cysteine at their N terminus, which has been added for conjugating them to a carrier protein for the immunization

Peptide name	Sequences	Location	Serum from rabbit
MXR P1	**C**VGTQFIR	178–184	#65158, #64541
MXR P2	**C**LAEIYVNSSFYK	332–343	#64795, #64850
MXR P3	**C**EISYTTSFCHQLR	366–378	#64795, #64850
MXR P4	**C**LFIHYISGYYR	454–465	#64853, #64851
MXR P5	**C**NDSTGIQNR	418–426	#64853, #64851
			
MRP1 P1	**C**PSDLLQQR	1511–1518	#A0151, #A0152
MRP1 P2	**C**DLWSLNK	240–246	#A0151, #A0152
			
NaK ATPase P1	**C**IPFNSTNK	478–485	#64845, #64846
NaK ATPase P2	**C**PTTPEWVK	74–81	#64845, #64846
NaK ATPase P3	**C**TGTLTQNR	368–375	#64855, #64856
NaK ATPase P4	**C**YEPAAVSE	10–17	#64855, #64856

**Figure 1:**
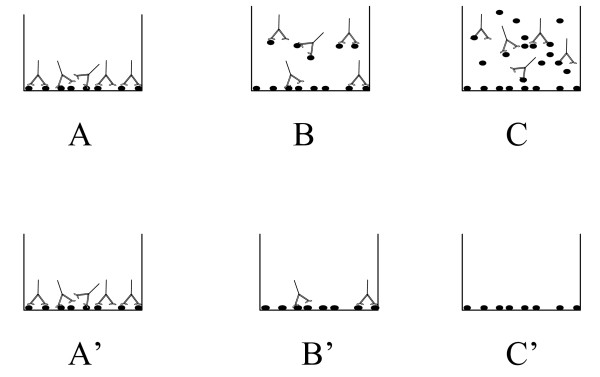
**The procedure of the 'Peptidomatrix' assay**. The ELISA Peptidomatrix is a competition assay: Peptides are bound to the plastic wells and they are subsequently exposed to antibodies generated against them. A labeled secondary antibody is used to detect the bound primary antibodies. In the assay itself the antibodies are mixed with soluble synthetic peptide in known amounts, to generate a calibration curve. The peptide in solution competes with the bound peptide and will affect the signal. As shown schematically in the figure: Panel A, A': No free peptide is added, thus the signal will be the highest. Panel B, B': A small amount of free peptide is added and the signal will be intermediate. Panel C, C': A large amount of competing peptide is added, thus no antibody binds to the well and the signal is minimal. (see Materials and Methods for details)

The Peptidomatrix assay, as described in the Introduction, is a competition assay (Figure 1) :Peptides are bound to plastic wells. Antibodies specific to the peptide are then added in solution and allowed to bind to the attached peptide in the presence or the absence of a sample digest. A calibration curve is generated in parallel with known quantities of free synthetic peptide. The concentration of soluble peptide in the sample is then measured by interpolation.

The assay will be most sensitive when the added peptide blocks the reaction between antibody and bound peptide at the lowest possible concentration. On the other hand, we need to obtain the highest possible signal-to-noise ratio in the final ELISA assay. To explore the optimal conditions for this, we performed a series of experiments in which we varied the amount of bound peptide and the concentration of antibody, before performing the competition with added peptide.

**Figure 2:**
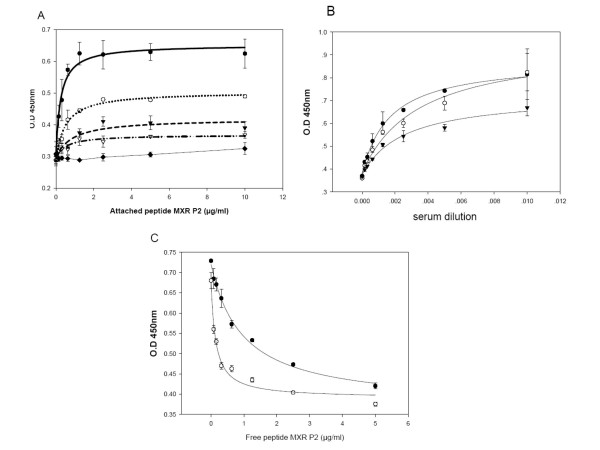
**Determination of the optimal conditions for the Peptidomatrix, using the peptide MXR P2 and the serum from rabbit # 64850**. (A) Binding of the antibody (at different dilutions) to wells containing increasing amounts of peptide. The actual amount of bound peptide has not been determined and it is assumed that it is proportional to the concentration used for coating the wells. Antibody dilutions: 1:100 (●), 1:200 (○), 1:1000 (▼), 1:2000 (▽) and pre-immune serum 1:100 (◆). (B) Titration of bound peptide with increasing concentrations of antibody (expressed as dilutions of the antiserum) 3 concentrations of attached peptide (1.0 (●) 0.5 (○) and 0.25 (▼) μg/ml) were used. (C) Competition between attached and free peptide using 2 concentration of attached peptide, 0.5 (●) and 0.25 (○) μg/ml, antibody dilution was 1:750. All the data were analyzed using equations 1 and 2 described in the methods. The, values of KdKd, Amax and D appear in Table 2. All the data points represent the average of triplicates.

**Figure 3:**
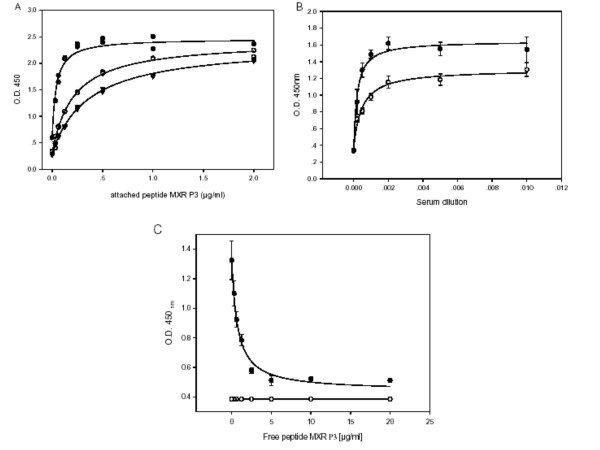
**Determination of the optimal conditions for the Peptidomatrix, using the peptide MXR P3 and the serum from rabbit # 64795**. Panels A, B and C are as described in the legend of Figure 2 except that in panel A there is no pre-immune serum reaction AND 1:2000, in panel B only two concentrations of attached peptide (0.5 (●) and 0.25(○)) were used and in panel C there is only one value of attached peptide (0.5 μg/ml), with the other curve being generated with pre-immune serum. The, values of KdKd, Amax and D appear in Table 3. All the data points represent the average of triplicates.

Fig 2A depicts the binding of polyclonal antibodies (raised against the peptide MXR P2), at four different dilutions, to peptide MXR P2 bound to the wells of the ELISA plate at increasing concentrations. The bound antibodies are detected by a secondary antibody (anti-rabbit IgG) conjugated to horseradish peroxidase. In Fig 3A, the same manipulations are performed with peptide MXR P3. In each case, the ELISA signal reaches a plateau as the amount of bound peptide is increased; the maximum size of the signal increases with increasing amount of antibody. The data were fitted (using Sigmaplot) to Equation 1 of Materials and Methods, a conventional hyperbolic isotherm, yielding the parameters of Amax (the maximum signal) and Kd (the concentration of bound peptide at which half the maximal increase in signal was obtained), together with a parameter D that represents the signal in the absence of bound peptide. Adding pre-immune serum at the highest concentration gave essentially no increase in signal above background (figs 2A). Figs 2B and 3B depict the signals obtained when we added increasing amounts of the relevant antibody at different fixed amounts of bound peptides MXR P2 and MXR P3. Again, the data for all these figures were fitted using Equation 1 of Materials and Methods, with the kinetic parameters listed in Table 2.

**Table 2:** Kinetic parameters obtained from the optimization of the Peptidomatrix conditions for peptide MXR P2 and the serum from rabbit # 64850 (see Figure 2) (± SD, n = 3).

A : increasing attached peptide at four fixed levels of added antibody				
	1 ^st^AB Concentration 0.0005 serum/PBS	1 ^st^AB Concentration 0.001 serum/PBS	1 ^st^AB Concentration 0.005 serum/PBS	1 ^st^AB Concentration 0.01 serum/PBS

Kd (μg/ml)	0.49 ± 0.25	0.89 ± 0.56	0.49 ± 0.097	0.24 ± 0.06
Amax (OD units)	0.076 ± 0.01	0.12 ± 0.01	0.22 ± 0.012	0.35 ± 0.024
D (OD units)	0.29 ± 0.008	0.3 ± 0.015	0.29 ± 0.009	0.3 ± 0.02

B: increasing added antibody at three fixed levels of attached peptide				

	Attached peptide 0.25 μg/ml	Attached peptide 0.5 μg/ml	Attached peptide 1 μg/ml	

Kd (serum dilutions)	0.003 ± 0.0007	0.003 ± 0.001	0.001 ± 0.0002	
Amax (OD units)	0.35 ± 0.023	0.56 ± 0.06	0.49 ± 0.02	
D (OD units)	0.38 ± 0.01	0.38 ± 0.02	0.38 ± 0.01	

C: Competition assay – Kd determined when increasing added free peptide at two fixed levels of attached peptide				

	Attached peptide 0.25 μg/ml	Attached peptide 0.5 μg/ml		

Kd (μg/ml)	0.14 ± 0.03	0.97 ± 0.15		
Amax (OD units)	0.29 ± 0.019	0.37 ± 0.016		
D (OD units)	0.39 ± 0.01	0.35 ± 0.01		

Figs 2C and 3C depict competition assays presented as the signals obtained when we chose a single concentration of added antibody and titrated it with increasing concentrations of added free peptide, at two concentrations of bound peptide in Fig 2 but at a single concentration in Fig 3, with the addition of a titration using a non-related peptide depicted in Fig 3C.

**Figure 4:**
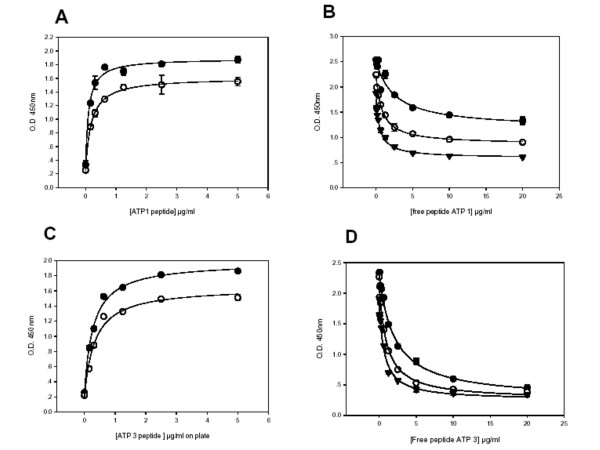
**Binding of the antibody (at different dilutions) to wells containing increasing amounts of ATPase peptide**. In 4A and 4C a different peptide/antibody combination is shown at two different dilutions of the antibody: (●) 1:1500; (○) 1:3000. Panel A: ATP1/RS64845; panel C ATP3/RS64855. Competition between attached and free peptide is depicted in B and D using 3 (dilutions of antibody: (1:400 (●); 1:800 (○); 1:1600 (▼). Peptide/antibody combinations are as in A and C, respectively for B and D. All the data points represent the average of triplicates.

Figure 4 depicts an analysis of the Peptidomatrix conditions for the detection of the Na,K-ATPase. Panels A and C show a titration using increasing concentrations of bound peptides ATP P1 and ATP P3, at two different concentrations of antibody. The parameters obtained by curve fitting of these data are recorded in Table 3. Panels B and D of Fig 4 depict inhibition curves, as in figures 2C and 3C, using the peptides chosen for the Na,K-ATPase, at different concentrations of the appropriate antibody, with parameters recorded in Table 3.

**Table 3:** Kinetic parameters obtained from the optimization of the Peptidomatrix conditions with the peptides and antibodies specific to Na/K ATPase (see figure 4) (± SD, n = 3)

A: increasing attached peptide at two fixed levels of added antibody			
	1 ^st^AB Concentration 0.00033 serum/PBS	1 ^st^AB Concentration 0.00066 serum/PBS	

Kd (μg/ml)	0.19 ± 0.01	0.1 ± 0.02	
Amax (OD units)	1.36 ± 0.02	1.56 ± 0.08	
D (OD units)	0.25 ± 0.02	0.38 ± 0.07	

B: Competition assay – Kd determined when increasing added free peptide at three fixed levels of added antibody			

	1 ^st^AB concentration 0.000625 serum/PBS	1 ^st^AB concentration 0.000125serum/PBS	1 ^st^AB concentration 0.0025 serum/PBS

Kd (μg/ml)	0.55 ± 0.1	0.83 ± 0.12	2.55 ± 1.21
Amax (OD units)	1.16 ± 0.05	1.35 ± 0.05	1.33 ± 0.17
D (OD units)	0.59 ± 0.04	0.86 ± 0.04	1.17 ± 0.18

C: increasing attached peptide at two fixed levels of added antibody			

	1 ^st^AB concentration 0.00033 serum/PBS	1 ^st^AB concentration 0.00067 serum/PBS	

Kd (serum dilutions)	0.33 ± 0.08	0.29 ± 0.04	
Amax (OD units)	1.46 ± 0.09	1.74 ± 0.06	
D (OD units)	0.19 ± 0.079	0.24 ± 0.05	

D: Competition assay- Kd determined when increasing added free peptide at three fixed levels of added antibody			

	1 ^st^AB concentration 0.000625 serum/PBS	1 ^st^AB concentration 0.00125 serum/PBS	1 ^st^AB concentration 0.0025 serum/PBS

Kd (μg/ml)	0.68 ± 0.12	0.99 ± 0.19	2.04 ± 0.3
Amax (OD units)	1.58 ± 0.06	1.87 ± 0.09	2.05 ± 0.08
D (OD units)	0.25 ± 0.05	0.25 ± 0.08	0.28 ± 0.08

Inspection of these figures and Tables shows that the received signal increases with increasing concentrations of either the bound peptide or the appropriate antibody until it reaches a plateau. Comparing the binding curves at increasing amounts of bound peptide or increasing concentrations of antibody we see that the derived Amax, the overall amplitude of the signal, increases accordingly. All this is expected, since increasing either the amount of binding sites or the concentration of the free ligand will increase the signal, until a maximum, and therefore saturation, is reached.

With regard to the inhibition (that is, competition) curves there is a similar trend, with Amax (the signal in the absence of externally added peptide) again increasing as function of either the attached peptide or the concentration of the antibody. Also this is expected, since Amax is the expression of the maximal binding of the antibody to the peptide, when there is no competition.

It is not immediately obvious, however, whether the values of the half-saturation concentrations, Kd, in the various experimental situations, should increase, decrease or be invariant. Table 4 summarizes the results of all the experiments using the MXR, ATPase and MRP1 peptides in terms of the trends of increase or decrease in the values of Amax and Kd. As expected in all cases, the parameter Amax increases as either the amount of bound peptide or of added antibody is increased. However, in most cases in Columns A (when it is the amount of pre-attached peptide that is continuously varied at fixed amounts of added antibody) and in Column B (antibody varied at fixed attached peptide), the Kd parameter decreases as the amount of bound peptide or of added antibody is raised. In the case of the competition experiments (where it is the concentration of free added peptide that is the variable) Amax again increases with an increase in the amount of bound peptide or of added antibody, but the Kd value increases in both the two conditions (Columns C and D).

**Table 4:** summary of the results with the different peptides.

	A- peptide titration		B- serum titration		C-competition (peptide titration)		D-competition (Antibody titration)	
	Kp	Amax	Ka	Amax	Kd	Amax	Kd	Amax

1	↓	↑	↓	↑	↑	↑		
2	↓	↑	↓	↑	↑	↑		
3	↓	↑	↔	↑	↑	↑		
4	↓	↑	↓	↑	↑	↑		
5	↓	↑	↓	↑				
6	↓	↑			↑	↑	↑	↑
7	↓	↑					↑	↑
8	↓	↑					↑	↑
9	↓	↑					↑	↑
10					↑	↑	↑	↑
11	↓	↑			↑	↑	↑	↑

A-the effect of increasing the amount of antibody.

B- the effect of increasing the amount of attached peptide.

C- the effect of increasing the amount of attached peptide on the parameters of the competition.

D- the effect of increasing the amount of antibody on the parameters of the competition.

(1)MXR P5/#853, (2) MXR P5/#851, (3) MXR P2/#850, (4) MXR P2/#795, (5) MXR P3/#795, (6) MXR P1/#841, (7) ATP P1/#840, (8) ATP P3/#855, (9) ATP P4/# 485, (10) MRP P1/#151 #152,(11) MRP P2/#151 & #152

We attempted to account for these trends in the Amax and Kd data by devising a theoretical equation the takes into account the binding of the peptides to the plastic substratum, the binding of the antibody to the bound peptide and, finally, the interaction between the free peptide and the antibody in the competition experiments. The derivation of the appropriate equations is given in the Appendix.

The binding of the antibody to the attached peptide is described by equation A2



**Figure 5:**
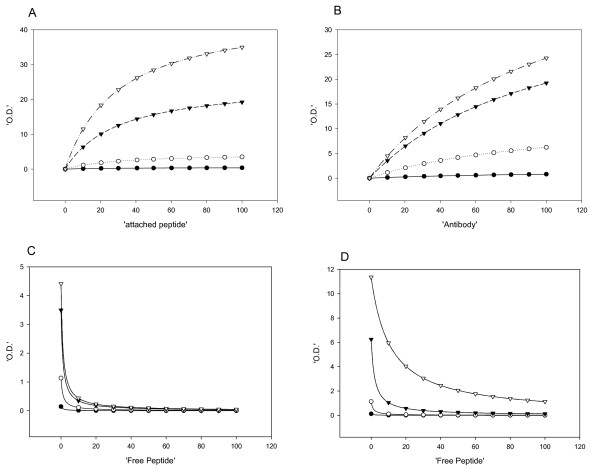
**Theoretical determination of the optimal conditions for the Peptidomatrix**. (A) Binding of the antibody (at different dilutions) to wells containing increasing amounts of peptide,0.1(●),1(○), 10(▼), 100(▽). (B) Titration of bound peptide (different amounts) with increasing concentrations of antibody, 0.1(●), 1(○), 10(▼), 100(▽). (C) Competition between attached and free peptide using 4 concentrations of attached peptide,0.1(●),1(○), 10(▼), 100(▽), first antibody 'dilution' was 10. (D) Competition between attached and free peptide using 4 concentrations of antibody 0.1(●),1(○), 10(▼), 100(▽), 'attached peptide' was 10. All these curves are simulations and were drawn using equation A2 (for panel A and B) and A5 (for panel C and D), with kinetic parameters of Ka = 30, Kp = 10, cap = 50 and Kf = 1, see the appendix for details. All the data were analyzed using the Michaelis-Menten equation (normal and descending hyperbola). The derived values of Kd and Amax appear in table 5.



This equation predicts the signal obtained when an amount P of bound peptide is added to a well having a maximal binding capacity of *cap *and a half-saturation concentration for this binding of *Kp*, where the concentration of added antibody is A, with a half-saturation concentration for the peptide/antibody isotherm of *Ka*. Fig 5 depicts the predictions of this equation when, in panel A, the concentration of bound peptide is varied continuously while that of the added antibody is set at different values. In panel B the antibody concentration varies continuously while the amount of bound peptide is varied stepwise. Arbitrary values were assigned to the other constants. These theoretical lines were fitted using equation 1 of the Materials and Methods section to obtain the parameters listed in sections A and B of Table 5. As expected, the Amax values increase with an increase in either bound peptide or added antibody. The Kd values are, however, invariant under these changes, in contrast to what was found experimentally. Figs 5C and 5D depict the theoretical predictions of the appropriate equation (equation A5) for the competition protocol, where it is now free peptide that is added with the added antibody (at varying concentrations of bound peptide and added antibody, Figs 5C and 5D, respectively).

**Table 5:** Kinetic parameters obtained by simulating the conditions for the Peptidomatrix using equations A2 and A5 (single binding site). Data in Fig 5.

A : increasing attached peptide at four fixed levels of added antibody				
	Antibody 0.1	Antibody 1	Antibody 10	Antibody 100

Kd	30.00	30.00	30.00	30.00
Amax	0.5	4.55	25.00	45.45

B: increasing added antibody at four fixed levels of attached peptide				

	Attached peptide 0.1	Attached peptide 1	Attached peptide 10	Attached peptide 100

Kd	100.00	100.00	100.00	100.00
Amax	1.61	12.50	38.46	48.54

C: Competition assay – Kd determined when increasing added free peptide at four fixed levels of attached peptide				

	Attached peptide 0.1	Attached peptide 1	Attached peptide 10	Attached peptide 100

Kd	1.10	1.10	1.10	1.10
Amax	0.15	1.14	3.5	4.41

D: Competition assay – Kd determined when increasing free peptide at four fixed levels of added antibody				

	Antibody 0.1	Antibody 1	Antibody 10	Antibody 100

Kd	1.01	1.10	2.00	11.00
Amax	0.12	1.14	6.25	11.36

Where we write  (see the appendix for details). Fitting these lines to equation 2 of Materials and Methods yields the parameters listed in sections C and D of Table 5. In both cases, the parameter Amax increases, as found experimentally. The Kd value, however, is again invariant as the amount of bound peptide is increased (in contrast to the experimental findings), but increases as the amount of added antibody increases (as was found experimentally).

In an attempt to resolve the discrepancy between the experimental data and the model predictions, we analyzed a more complex model in which the plastic substratum bears at least two classes of sites to which the peptide can bind. The two classes have different affinities for peptide (*Kp*_1 _and *Kp*_2_) and the bound peptide then displays a different affinity for a particular antibody (*Ka*_1 _and *Ka*_2_). (We envisage this as depicted in the diagram of Fig 7, where a peptide is shown as binding at one end only to the substratum or at both ends, and the antibody can then bind with a higher or a lower affinity to the peptide.) The free peptide in the solution binds, of course, with a single affinity *Kf*, since it binds to the unbound antibody. The equations describing this more complex model are equation A6 for the binding curves and equation A7 for the competition curves.





**Figure 6:**
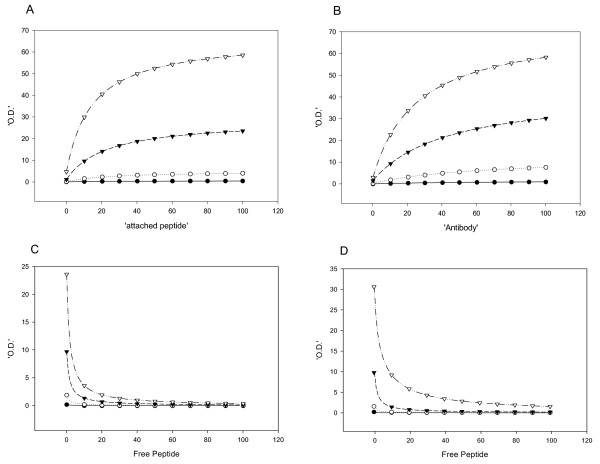
**Theoretical determination of the optimal conditions for the Peptidomatrix ACORDING TO 2 SITES**. (see the appendix for details). Panels A, B, C and D are as described in the legend of Figure 5 except that the equations used in this case were A6 (for panel A and B) and A7 (for panel C and D). All data were analyzed using the Michaelis-Menten equation (normal and descending hyperbola), using the parameters Ka1 = 30, Ka2 = 3, Kp1 = 10, Kp2 = 100, cap = 50 and Kf = 1. The derived values of Kd and Amax appear in table 6.

The derivation of these equations can be found in the Appendix. The predictions of the equations are depicted in Fig 6A and 6B (for equation A6) and C and D (for equation A7) and recorded in Table 6. As expected, the values of Amax increase when either the amount of bound peptide or of added antibody increases, but now the value of Kd *falls *substantially with an increase in these inputs. This is the result found experimentally as listed in Table 4. For the competition protocol, the theoretical predictions are now of an increase in the half saturation concentration for added free peptide when either bound peptide or added antibody is increased. (The effect is small but consistent, when it is the attached peptide that is varied). This is in accord with the experimental data, but is in contrast with the predictions of the simple one-site model, in the case where it is the attached peptide's concentration that is increased.

**Figure 7:**
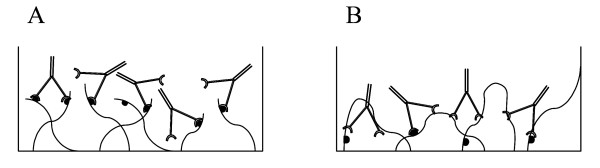
**Reciprocal relationship of peptide-solid support binding and antibody-peptide binding**. Our data indicate that when the peptide binds to the plate tightly, with a low Kp, the antibodies bind the peptide more loosely, with high Kd. And, vice versa, if the peptide is bound loosely, then the antibody-peptide bond will be stronger. This figure represents schematically a possible mechanism for this phenomenon. Panel A: The peptide binds with low affinity, represented here with a single attachment point to the solid support. As a result the antibody has facilitated access to the peptide and the peptide -antibody bond is strong. Panel B: The peptide binds tightly, represented here as multiple attachment points. In this case the access of the antibodies is hindered by steric interference and the antibody-peptide bond is weaker. It is worth noting that our results indicate that by changing the concentration of the peptide at the time of plating it is possible to manipulate the strength of the attachment: High concentration of the peptide result in low affinity binding while low concentrations of the peptide result in high affinity binding.

**Table 6:** Kinetic parameters obtained by simulating the conditions for the Peptidomatrix, using equations A2 and A7 (two binding sites). Data in Fig 6.

A: increasing attached peptide at four fixed levels of added antibody				
	Antibody 0.1	Antibody 1	Antibody 10	Antibody 100

Kd	25.53	25.22	22.69	14.70
Amax	0.52	4.80	27.49	61.77

B: increasing added antibody at four fixed levels of attached peptide				

	Attached peptide 0.1	Attached peptide 1	Attached peptide 10	Attached peptide 100

Kd	70.55	65.62	43.28	25.68
Amax	1.58	12.28	41.07	69.42

C: Competition assay – Kd determined when increasing added free peptide at four fixed levels of attached peptide				

	Attached peptide 0.1	Attached peptide 1	Attached peptide 10	Attached peptide 100

Kd	1.3869	1.4333	1.6399	1.8080
Amax	0.2291	1.9375	9.7190	23.5973

D: Competition assay – Kd determined when increasing free peptide at four fixed levels of added antibody				

	Antibody 0.1	Antibody 1	Antibody 10	Antibody 100

Kd	1.001	1.01	1.64	4.12
Amax	0.16	1.52	9.72	28.98

## Discussion

We undertook these experiments in order to ascertain what might be the most optimal conditions for performing the Peptidomatrix assays. Our aim was to find conditions that would give us the maximum sensitivity (i.e. enable us to detect the smallest possible amount of target protein) and yet the highest possible signal-to-noise ration. The Peptidomatrix assay is done using the competition protocol depicted in Fig 1, experimental results with this protocol being given in Figs 2C, 3C, 4B and 4D and in the Supplementary materials. It is clear that, both in theory and in practice, the highest signal-to-noise ratio is obtained by using plateau values of the bound peptide and of the added antibody. But are these the conditions that will give the maximum sensitivity?

Assuming that the peptide binds to the plastic with one modality and that the bound peptide displays one single site to the antibody (one-site binding model) we would predict (Table 5, sections C and D) that the half-saturation concentration for competition by added free peptide in the competition assay would be invariant with respect to amount of bound peptide, but would increase with the concentration of added antibody. A low Kd in the competition assay is the condition for maximum sensitivity, since, in this case, a small amount of externally added peptide can be detected. Thus, the condition of maximum attached peptide and a low added antibody would be the most sensitive situation, yet would give a good signal to noise ratio. But does this one-site model give a correct explanation of the data in a real situation? Experimentally, we find that increasing the amount of attached peptide leads to an apparent higher affinity of antibody to this bound peptide, while increasing the amount of added antibody again leads to higher apparent affinities for the attached peptide. This is in contrast to the predictions of the one site model. Solving the two-site model indeed leads to predictions that more closely model the real situation but, happily, still leave us with a similar recommendation for the conditions that give maximum sensitivity and yet a high signal to noise ratio. Increasing the amount of pre-attached peptide does now lead to an increase in the Kd for competition by added peptide, but this increase is small. Increasing the amount of added antibody increases the half-saturation concentration for the free peptide, and hence decrease the sensitivity of the assay, yet this effect is smaller for the two site model than for the one-site model. Maximum sensitivity will be found when one uses the maximum possible coating by pre-added peptide and a minimum amount of added antibody, yet enough to give an easily measurable signal.

A model that assumes that the peptide binds to the plastic substrate with at least two modalitiesand at least two different binding affinities for the antibody (the two-site model) agrees with the experimental findings. According to this simulation, the peptide molecules that are bound with the highest affinity and hence are bound first, i.e., at the lowest concentrations of added peptide, are those to which the antibody binds with *lowest *avidity. Thus, as the amount of bound peptide increases, the half-saturation concentration of the attached peptide for antibody substantially decreases (its avidity increases), and the added free peptide is less able to compete. But this effect is not very large and four orders of magnitude of increasing attached peptide raise the Kd for the competing added peptide by less than 50% (Table 6, section C). On the other hand, at a fixed amount of attached peptide, raising the amount of added antibody by a similar four orders of magnitude raises the Kd for the competing peptide four fold. Thus, to obtain the highest sensitivity, together with the highest signal-to-noise ratio, it would appear to be preferable to reduce as far as possible the concentration of the antibody and to increase, rather, the amount of bound peptide.

We believe that this phenomenon of the binding in several possible conformations is a universal phenomenon for binding of small peptides to plastic substratum and we have to take this into consideration when we design such an assay.

Nothing in our analysis rules out the possibility that there is a continuous distribution of affinities between the substratum and the added peptide in the binding isotherm. We can only state that at least two classes of sites seem to be present.

## Conclusion

We have conducted a study aimed at the optimization of the Peptidomatrix and developed a theoretical model that explains the experimental data. This model takes into account the interaction of the peptides with the ELISA plate, the interactions of the antibodies with the bound and the free peptides and the competition between them.

## Methods

### Materials

Polyclonal antibodies were custom made by Affinity Bioreagents (ABR, Golden, CO)

Peptides: Synthetic peptides were obtained by a number of sources, ABR, Biosight Ltd (Karmiel, Israel) and the service department of the Hebrew University in Jerusalem

### Peptide selection

The peptides on which the ELISA is based are chosen using a three-fold screening process: First, a list is made of all the peptides that are likely to be present in a tryptic digest of the protein to be identified. The sequences of the desired proteins were retrieved from the NCBI databases. A virtual "digest" was performed on the sequence using the Microsoft Word program. Next, peptides 7–15 amino acid long were selected. Each peptide from this selection was checked for its uniqueness amongst all the polypeptide chains that comprise the human proteome, using the BLAST program (parameters: PAM 30 Gap Costs Existence 5, Extension 2). From this limited list, we chose the more hydrophilic peptides as being those that would be most likely to be good antigens [6,13]. A number of these selected peptides were ultimately chosen for production of antibodies, and then used in the protocol described below

The peptides chosen for this investigation are shown in Table 1.

### Polyclonal antibodies

The polyclonal antibodies used throughout this study were generated in rabbits by Affinity Bioreagents, Inc. (ABR, Golden, CO), whole serum was used in all the experiments. The antigen for immunization was prepared also by ABR, including the synthesis of the peptides, the conjugation to a carrier and the injection to rabbits. The rabbits were bled once before immunization and 3 or 4 times after immunization. The titers were recorded and the various bleedings kept and used for the development of the immunoassay.

### Competitive ELISA

Maxisorp ELISA plates (NUNC, Denmark) were coated overnight at 4°C, with 0.1–2 μg/ml of the relevant synthetic peptide in 0.1 M carbonate buffer pH 9.6 and blocked 2 hours at room temperature with a blocking buffer containing 3% BSA/0.05 % Tween 20 in phosphate buffered saline (PBS, 0.1 M phosphate buffer, 150 mM NaCl, pH 7.2). For the binding experiments the immune serum was diluted at the desired concentration in blocking buffer. For the competition experiments serial binary dilutions of the synthetic peptide (from 5 to 0.078 μg/ml) and a blank sample were prepared in blocking buffer containing the desired antibody at the desired concentration. 100 μl of these solutions were added to the wells and incubated at room temperature 1–3 hours. The wells were washed 4 times with 1× PBS/0.05% Tween 20 and 100 μl of horseradish peroxidase(HRP)-conjugated 2^nd ^Ab (anti-rabbit) diluted 1:10,000 or 1:20,000 in blocking buffer were added in each well. After incubation for one hour at RT and washing as above, the bound HRP conjugate was detected by adding 100 μl of tetramethyl benzidine (TMB). The peroxidase reaction was stopped after 5- minutes by the addition of 50 μl 0.5 M H_2_SO_4_. Optical densities at 450 nm were measured using an ELISA reader. The assay was in triplicates.

### Data elaboration

The data from the ELISA plate reader are fed into a data analysis template in the program Sigmaplot (SPSS, Chicago, IL). A plot of the OD450 vs. the concentration of the antibody or the concentration of the free peptide is drawn. The plot is then fitted, using a regression program, to a hyperbola fitting an ascending (Equation. 1) or a descending (Equation. 2) hyperbolic 3-parameter equation, as described in the text:

Equation 1 

Equation 2 

where Amax is the calculated maximal amplitude of the curve, D the predicted minimum of the ELISA readings, corresponding essentially to the background signal, S the concentration of the antibody or the attached peptide (in equation 1) or the free peptide (in equation 2) and Kd is the concentration that gives one-half of the shift between maximum and minimum readings.

## Appendix

Derivation of the equations that describe the competition between bound and free peptide for the appropriate antibody:

The first equation describes the binding between the peptide and the plastic substratum. When an amount *P *of the peptide that is to be bound is added to a well having a maximal binding capacity of *cap *and a half-saturation concentration for this binding of *Kp*, we obtain for the amount of bound peptide, BP, according to a simple hyperbola, the equation.:



The second equation describes the binding, again described by a hyperbola, between the bound peptide and the added antibody, where the concentration of free added antibody is A, with a half-saturation concentration for the attached peptide/antibody isotherm of *Ka*. The term *signal *is the ELISA signal obtained, a measure of the antibody that is bound to the peptide that is itself bound to the plastic substratum.



To describe the competition between attached and free peptide we have to use 3 binding parameters. The first and the second are those we used above to describe the titration, the third parameter is the affinity between the free peptide and the free antibody (we assume that the affinities of the antibody for the free and for the attached peptides are different). With a half-saturation concentration for the free peptide/antibody isotherm of *Kf*, a concentration of added free peptide as *F*, and a concentration *TotA *of total antibody, we have (were *A *is now the concentration of *free *added antibody:



Substituting from A3 into A2, we obtain the equation that describes the competition as:



which follows the Hyperbolic Decay equation on the variable *F*.

Substituting from A1, and simplifying, we obtain:



The predictions of these equations are given in the Results section. When we compared these theoretical predictions with the experimental results, we found a lack of agreement between them. Thus, we explored the possibility that we have at least 2 sites of binding between the peptide and the plastic substratum (two different values for *Kp*), and, respectively, two different values for *Ka*. Expanding Equation A2 and A4 to include the presence of two binding sites for the peptide to the plastic substratum, and two affinities for these forms of bound peptide to the antibody, we have:



And we obtain for the equation of the competition:



## Competing interests

The author(s) declare that they have no competing interests.

## Authors' contributions

HG was the first to develop the Peptidomatrix procedure. J B-S further developed the procedure. OB and HG performed most of the experimental work described in this paper. OB wrote the manuscript and developed the model of the two conformations of the peptide. UP provided intellectual guidance and aided in the preparation of the manuscript. WDS initiated the project, developed the mathematical analysis, directed the research and aided preparation of the manuscript.

## References

[B1] Andersson K, Choulier L, Hamalainen MD, Van Regenmortel MH, Altschuh D, Malmqvist M (2001). Predicting the kinetics of peptide-antibody interactions using a multivariate experimental design of sequence and chemical space. J Mol Recognit.

[B2] Choulier L, Andersson K, Hamalainen MD, Van Regenmortel MH, Malmqvist M, Altschuh D (2002). QSAR studies applied to the prediction of antigen-antibody interaction kinetics as measured by BIACORE. Protein Eng.

[B3] Crowther JR (2000). The ELISA guidebook. Methods Mol Biol.

[B4] Crowther JR (1995). ELISA. Theory and practice. Methods Mol Biol.

[B5] Friguet B, Djavadi-Ohaniance L, Goldberg ME (1989). Polypeptide-antibody binding mechanism: conformational adaptation investigated by equilibrium and kinetic analysis. Res Immunol.

[B6] Sela M (2000). Structural components responsible for peptide antigenicity. Appl Biochem Biotechnol.

[B7] Schwemmle M, Billich C (2004). The use of peptide arrays for the characterization of monospecific antibody repertoires from polyclonal sera of psychiatric patients suspected of infection by Borna Disease Virus. Mol Divers.

[B8] Rondard P, Goldberg ME, Bedouelle H (1997). Mutational analysis of an antigenic peptide shows recognition in a loop conformation. Biochemistry.

[B9] Kusnezow W, Hoheisel JD (2003). Solid supports for microarray immunoassays. J Mol Recognit.

[B10] Nieba L, Krebber A, Pluckthun A (1996). Competition BIAcore for measuring true affinities: large differences from values determined from binding kinetics. Anal Biochem.

[B11] Loomans EE, Gribnau TC, Bloemers HP, Schielen WJ (1998). Adsorption studies of tritium-labeled peptides on polystyrene surfaces. J Immunol Methods.

[B12] Harlow E, Lane D (1988). Antibody-antigen interaction. Antibodies: A laboratory manual CSH Laboratory Press; edition.

[B13] Van Regenmortel MH (2001). Antigenicity and immunogenicity of synthetic peptides. Biologicals.

